# Early Prediction of Cardiac Arrest Based on Time-Series Vital Signs Using Deep Learning: Retrospective Study

**DOI:** 10.2196/78484

**Published:** 2026-01-09

**Authors:** Yong Li, Lei Lv, Xia Wang

**Affiliations:** 1 College of Artificial Intelligence and Computer Science Northwest Normal University Lanzhou China; 2 Department of Pharmacy, the People’s Hospital of Gansu Province Lanzhou China

**Keywords:** cardiac arrest, prediction, electric medical record, vital signs, deep learning

## Abstract

**Background:**

Cardiac arrest (CA), characterized by an extremely high mortality rate, remains one of the most pressing global public health challenges. It not only causes a substantial strain on health care systems but also severely impacts individual health outcomes. Clinical evidence demonstrates that early identification of CA significantly reduced the mortality rate. However, the developed CA prediction models exhibit limitations such as low sensitivity and high false alarm rates. Moreover, issues with model generalization remain insufficiently addressed.

**Objective:**

The aim of this study was to develop a real-time prediction method based on clinical vital signs, using patient vital sign data from the past 2 hours to predict whether CA would occur within the next 1 hour at 5-minute intervals, thereby enabling timely and accurate prediction of CA events. Additionally, the eICU-CRD dataset was used for external validation to assess the model’s generalization capability.

**Methods:**

We reviewed and analyzed 4063 patients from the MIMIC-III waveform database, extracting 6 features to develop a deep learning–based CA prediction model named TrGRU. To further enhance performance, statistical features based on a sliding window were also constructed. The TrGRU model was developed using a combination of transformer and gated recurrent unit architectures. The primary evaluation metrics for the model included accuracy, sensitivity, area under the receiver operating characteristic curve (AUROC), and area under the precision-recall curve (AUPRC), with generalization capability validated using the eICU-CRD dataset.

**Results:**

The proposed model yielded an accuracy of 0.904, sensitivity of 0.859, AUROC of 0.957, and AUPRC of 0.949. The results showed that the predictive performance of TrGRU was superior to that of the models reported in previous studies. External validation using the eICU-CRD achieved a sensitivity of 0.813, an AUROC of 0.920, and an AUPRC of 0.848, indicating excellent generalization capability.

**Conclusions:**

The proposed model demonstrates high sensitivity and a low false-alarm rate, enabling clinical health care providers to predict CA events in a more timely and accurate manner. The adopted meta-learning approach effectively enhances the model’s generalization capability, showcasing its promising clinical application.

## Introduction

Cardiac arrest (CA) is characterized by the abrupt cessation of the heart’s blood-pumping function, marked by the absence of major arterial pulsations and heart sounds. This leads to severe ischemia and hypoxia in vital organs (eg, the brain), ultimately culminating in death. The most common cause of CA is ventricular fibrillation [1]. In the United States, there are approximately 209,000 cases of in-hospital CA each year, and the global survival rate is less than 25%. Previous studies have demonstrated that early recognition of CA can improve the survival rate within the first hour by approximately 29% and before hospital discharge by 19% [2]. Therefore, it is essential to develop a model that can reliably predict the occurrence of CA events.

In clinical practice, traditional assessment methods based on scoring, such as the Simplified Acute Physiology Score II, Sequential Organ Failure Assessment, and Modified Early Warning Score, have long served as crucial reference tools for clinicians to identify the risk of clinical deterioration and initiate timely interventions [3-5]. However, such scoring systems generally suffer from the dual limitations of low sensitivity and high false alarm rates. In recent years, machine learning has been applied extensively in health care data analysis, showing significantly better predictive performance than traditional scoring systems. Nevertheless, machine learning still faces challenges in analyzing high-dimensional and complex time-series data, which makes these approaches inadequate to meet the demands of current clinical practice.

Kim et al [6] developed a CA prediction model using the TabNet classifier to predict CA events. Their model achieved an area under the receiver operating characteristic curve (AUROC) of 0.79 on the MIMIC-IV dataset. Wu et al [7] developed a CA prediction model using extreme gradient boosting (XGBoost) based on 20 variables, including vital signs, laboratory results, and electrocardiogram reports. The model accuracy was 0.889, and the AUROC was 0.958. Similarly, Yijing et al [8] extracted vital sign data from the MIMIC-III database and used XGBoost to develop a model that achieved an accuracy of 0.96 and an AUROC of 0.94. An early warning model based on a recurrent neural network was proposed by Kwon et al [9], which yielded an area under the precision-recall curve (AUPRC) of 0.04 and an AUROC of 0.85.

Current methods based on deep learning and machine learning have demonstrated good performance in predicting CA events. However, they still suffer from low sensitivity and high false alarm rates as well as the inability to make real-time predictions. In particular, the method proposed by Lee et al [10] exhibited significant performance fluctuations when predicting CA events within 24 hours, a limitation that has also been demonstrated in previous studies. These shortcomings will lead to a substantial waste of medical resources and an increase in operational costs. As a result, current CA prediction methods face significant challenges in practical applications.

In this study, we propose TrGRU, a deep learning–based model that accurately predicts CA events within an hour using only 6 limited clinical vital signs without relying on patient laboratory test results. Meanwhile, we adopt a meta-learning framework, which significantly improves the adaptability of the model across different datasets and clinical settings [11,12].

## Methods

### Dataset

In this study, we used 2 databases: the MIMIC-III database [13] and eICU-CRD [14]. The MIMIC-III database, developed by the Massachusetts Institute of Technology Laboratory for Computational Physiology, contains data from 53,432 adult patients and 8100 neonatal patients admitted between 2001 and 2012. The database includes demographic data, vital signs, medications, laboratory measurements, physician orders, procedure codes, diagnosis codes, imaging reports, hospitalization duration, and survival data, among others.

The eICU-CRD is a multicenter intensive care unit (ICU) database that covers data from more than 200,000 ICU admissions across 208 hospitals in the United States between 2014 and 2015. The data were collected through Philips eICU program, which includes vital sign measurements, nursing documentation, severity of illness scores, diagnostic information, treatment details, and more.

We randomly allocated 70% (2844/4063) of the dataset for the training set to develop the model, with10% (406/4063) as the validation set to adjust and determine the hyperparameters of the model, and the remaining 20% (813/4063) as the test set to evaluate the model performance; this portion was not used in the model training.

### Ethical Considerations

This study used publicly available critical care databases, including the MIMIC-III and the eICU-CRD. Both databases received institutional review board approval from the hospitals that originally contributed the data, and informed consent was obtained at the time of data collection. All patient records were deidentified in accordance with the Health Insurance Portability and Accountability Act (HIPAA). Access to the databases was granted through the PhysioNet credentialing process. Therefore, according to institutional policies regarding secondary research using publicly deidentified data, additional ethics approval from our institution was not required.

### Problem Definition

**Figure 1:**
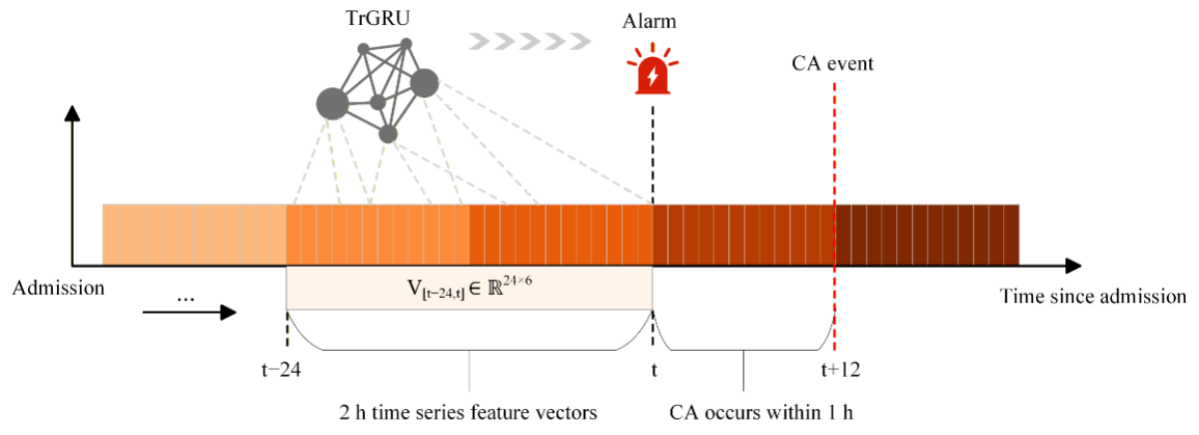
Cardiac arrest (CA) event monitoring process for the proposed TrGRU model.

As shown in Figure 1, let the given set of patients be P = {p_1_, p_2_, ..., p_n_} feature vector V*_t_* = [*hr, rr, sbp, dbp, map, spo2*]. V_t_ represents the vital sign monitoring data of patient p at time point t, including “heart rate” and “systolic blood pressure,” among others. These data were continuously collected during the hospitalization period T, T = {t_1_, t_2_, …, t_j_} and t_i_–_ti–j_ = 5 minutes. Then, 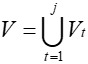 represents the multivariate time-series feature vectors of the patient at all time points during hospitalization. Therefore, the CA prediction task can be formally described as follows: given the multivariate time-series feature vector V for patient p in the hospitalization interval T, the goal of the prediction task is, for any time point t_i_∈T, to use the 24 multivariate time-series feature vectors from the time window (t–24 and t) to predict the probability of a CA event occurring within the time window (t and t+12) using the TrGRU model.

### Development Process

#### Review

**Figure 2:**
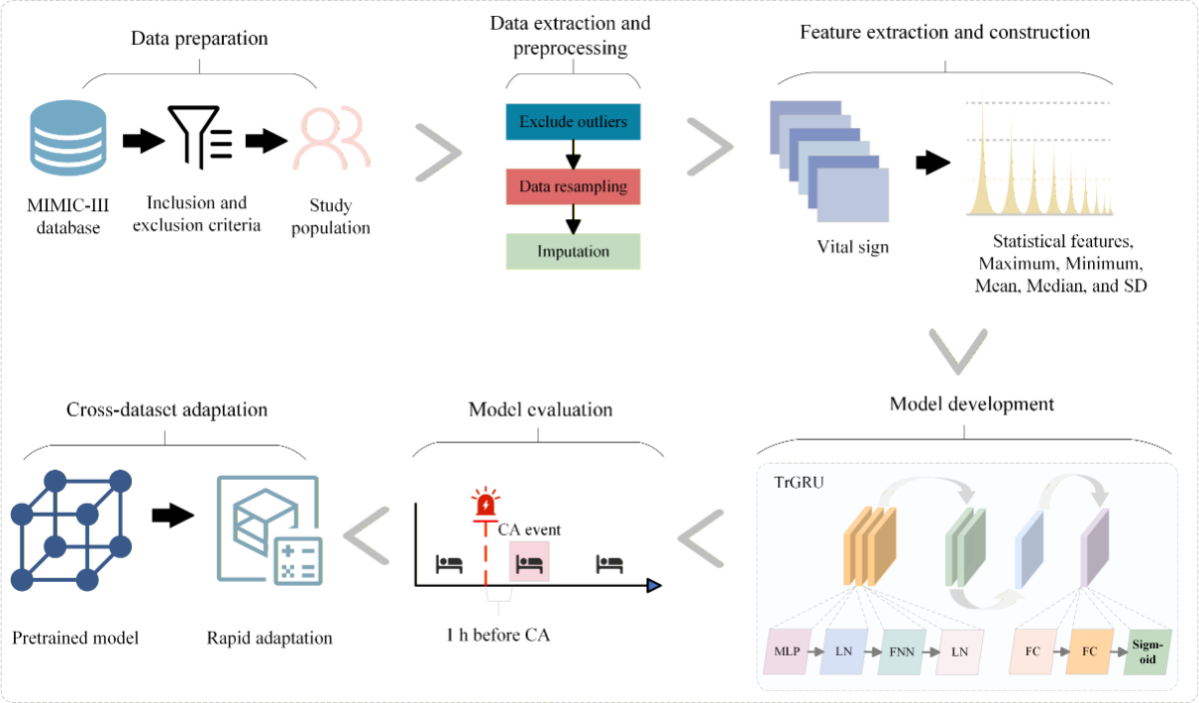
Development process of the TrGRU model. The development process of the TrGRU model consists of 6 steps. FC: fully connected layer; FNN: feedforward neural network; LN: layer normalization; MLP: multilayer perceptron.

As shown in Figure 2, the development process of the TrGRU model consisted of 6 steps: data preparation, data extraction and preprocessing, feature extraction and construction, model development, model evaluation, and cross-dataset adaptation.

#### Data Preparation

We extracted data from the MIMIC-III databases and eICU-CRD to construct a study cohort that met the inclusion and exclusion criteria. The patients in these 2 databases differ. The MIMIC-III database includes patients admitted to the ICU as well as some hospitalized patients not admitted to the ICU, while the eICU-CRD database contains only patients admitted to the ICU. The number of CA events for each patient also varies between the 2 databases. While each patient in the eICU-CRD frequently records several CA events, each patient in the MIMIC-III database typically only records one CA event. To increase the sample size of CA events, we treated multiple CA events experienced by a single patient as independent samples for model development.

**Figure 3:**
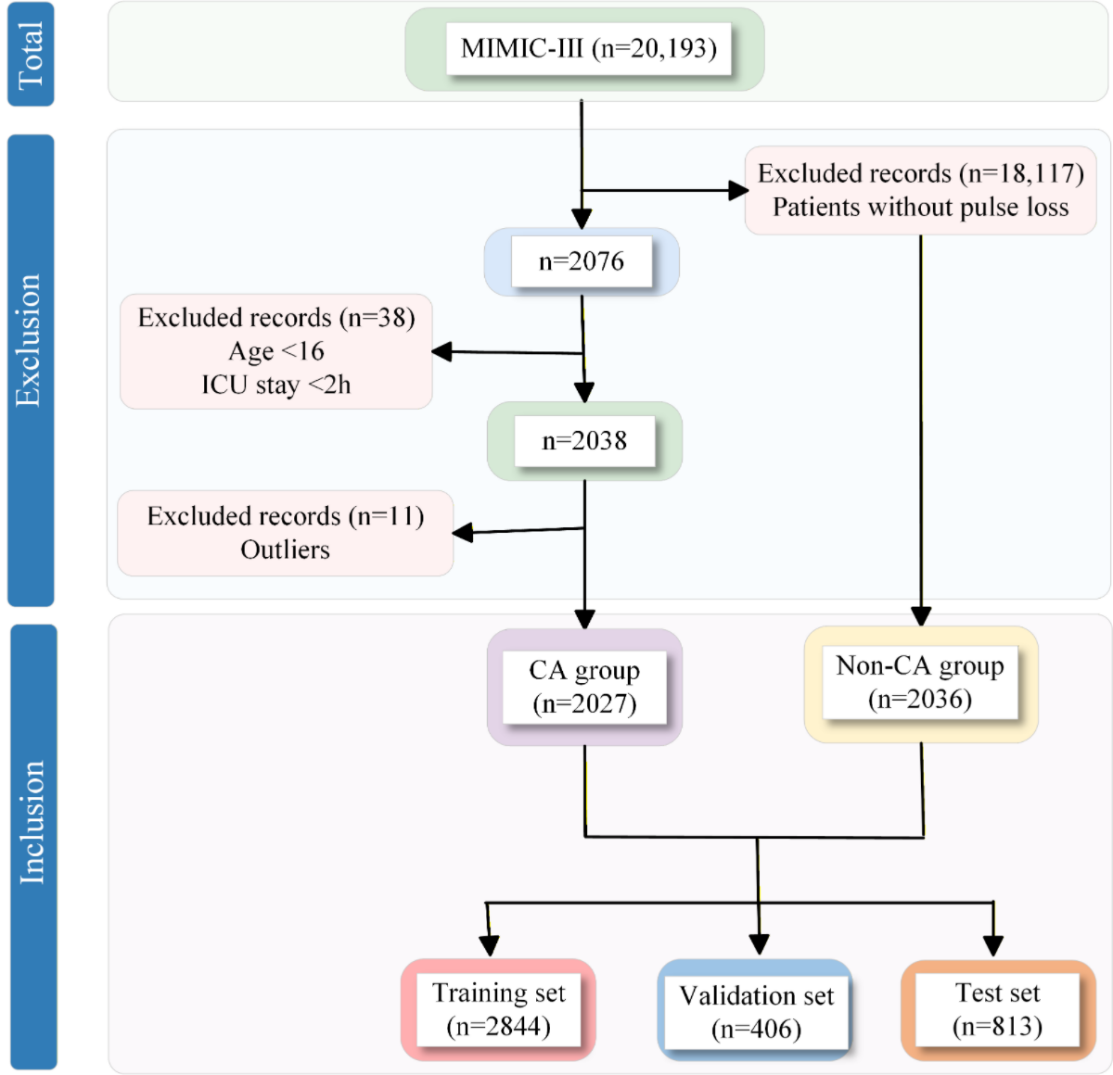
Patient inclusion and exclusion flow diagram for the MIMIC-III. CA: cardiac arrest; ICU: intensive care unit.

A series of criteria were applied during the inclusion and exclusion process of the MIMIC-III database to select the study cohort. As shown in Figure 3, 20,193 patients were included in this study, with 38 (0.19%) patients younger than 16 years and those who experienced CA within 2 hours of admission being excluded. In addition, 11 (0.05%) patients with outliers were excluded. CA is defined as the loss of a detectable pulse during attempts at resuscitation, and patients in this category were included in the CA group. Patients in the non-CA group were randomly selected from the remaining patients who did not experience a loss of pulse. Ultimately, the study population consisted of 4063 (20.12%) patients, with 2027 (49.89%) in the CA group and 2036 (50.11%) in the non-CA group of whom were not.

The inclusion and exclusion criteria applied to the eICU-CRD were the same as those used for the MIMIC-III database.

#### Data Extraction and Preprocessing

In this study, patient vital sign data were extracted from the MIMIC-III database, including heart rate (HR), respiratory rate, systolic blood pressure, diastolic blood pressure, mean arterial pressure, and oxygen saturation (SpO_2_). Due to the potential for data errors caused by equipment malfunction, the acceptable range for each vital sign value was determined based on the advice of medical experts. Data falling outside these ranges were considered outliers and excluded. Subsequently, the vital signs time-series data with a 1-minute sampling frequency were resampled to a 5-minute frequency. For time points without sampled values, these were marked as missing. We used median imputation to handle these missing values [15]. This method fills in missing values by identifying the median of the data, which is less affected by extreme values and thus provides a relatively stable substitute.

Additionally, because each feature is measured on a different scale, we applied minimum-maximum normalization to standardize them. Minimum-maximum normalization is a linear transformation method that maps data values to a fixed range. In this study, the values were scaled to the range (0,1). This process is achieved by scaling the minimum and maximum values of the data, ensuring that the transformed values are independent of the scale and range of the original data.

Although the number of patients included in the CA and non-CA groups was nearly equal, the samples input into the model were generated using a 2-hour sliding time window, and CA events occurred far less frequently than nonevents; that is, the number of positive samples was substantially lower than that of negative samples, resulting in class imbalance at the window level. Therefore, we mitigated the impact of class imbalance on model training by increasing the number of positive samples through oversampling and reducing the number of negative samples through undersampling.

#### Feature Extraction and Construction

We used 6 preprocessed vital signs as model features: HR, respiratory rate, systolic blood pressure, diastolic blood pressure, mean arterial pressure, and SpO_2_. These vital signs are widely used in clinical practice, and abnormal fluctuations in these measures are associated with the occurrence of CA. Previous studies also demonstrated that models developed based on these vital signs performed well in predicting CA events, making them suitable as model features [16,17].

In addition, we also created statistical features based on a sliding window. A fixed-length sliding window of 2 hours was applied, with a 5-minute time step, to segment each vital sign. The mean, median, minimum, maximum, and SD of each feature were then calculated for the time-series segments of the vital sign data within each window.

#### Model Development

We developed the TrGRU model based on deep learning methods, transformer [18] and gated recurrent unit (GRU) [19], using 2-hour time window vital sign data to predict CA events within the next 1 hour. By inputting the 2-hour data into the model, the risk score for a patient experiencing CA within the next 1 hour was evaluated. When the predicted score exceeded the risk threshold, it indicated a higher likelihood of the patient experiencing CA within 1 hour, and the patient was labeled as an event.

**Figure 4:**
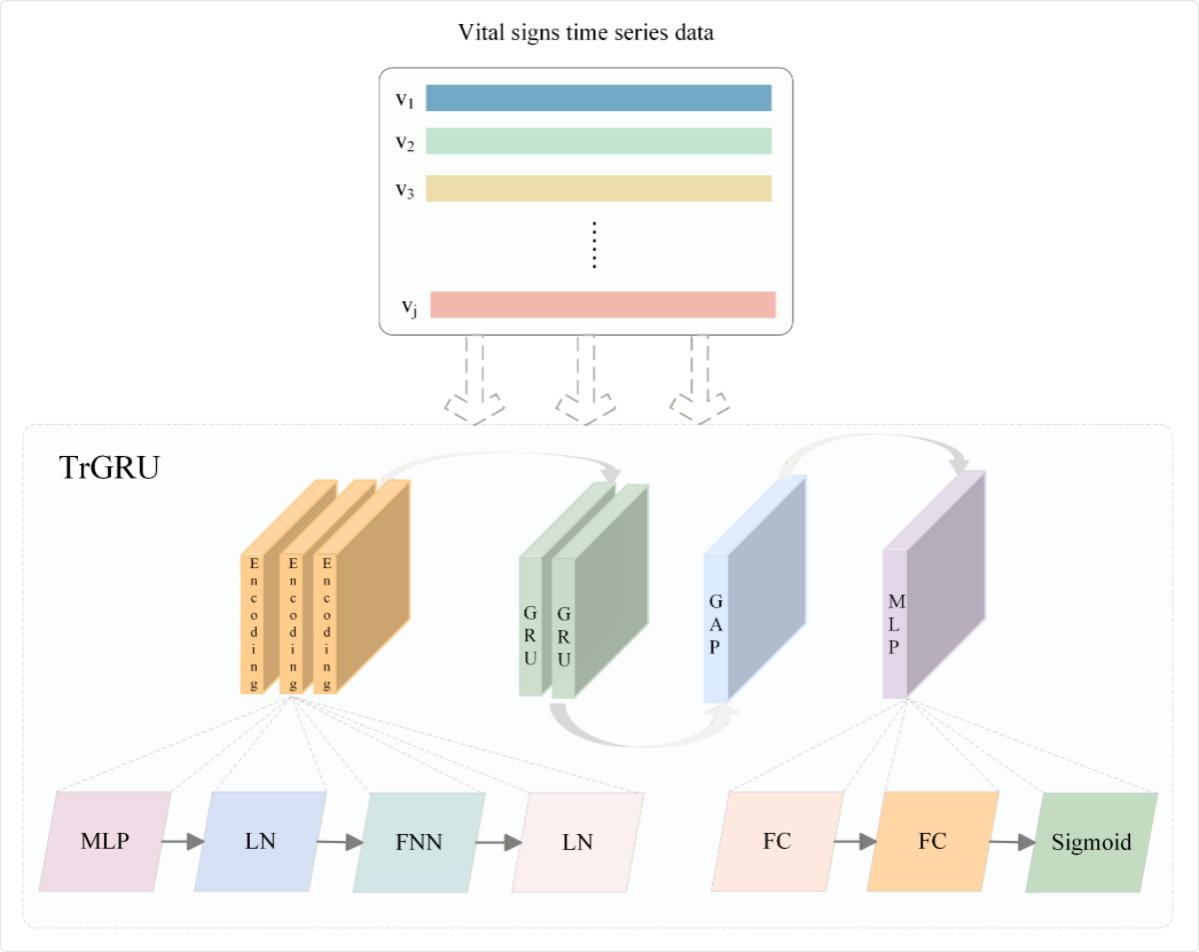
Architecture diagram of the TrGRU model. V1, V2, V3, and Vj are multivariate time-series feature vectors. GAP: global average pooling; GRU: gated recurrent unit; FC: fully connected layer; FNN: feedforward neural network; LN: layer normalization; MLP: multilayer perceptron.

As shown in Figure 4, the TrGRU is a hybrid model that combines a transformer and a GRU, designed to handle time-series data. Typically, the transformer is composed of an encoder and a decoder. In the TrGRU architecture, 3 encoder layers were stacked first, followed by 2 GRU layers. After the GRU layers, global average pooling is applied to compress the dimensionality of the time-series data, which pools the output feature vector of each sequence into a fixed-length vector. The pooled output was further processed by a multilayer perceptron head, in which the decoder was replaced with fully connected layers, as decoding was no longer necessary.

#### Model Evaluation

##### Baseline Models

To evaluate the performance of the proposed model, it was compared with 4 baseline models: random forest, logistic regression, XGBoost, and light gradient boosting method (LGBM).

##### Evaluation Metrics

We used accuracy, sensitivity, specificity, AUROC, AUPRC, and the false alarm rate to evaluate the performance of the model. AUROC is a metric that measures the performance of a binary classification model across different thresholds. The closer the AUROC value is to 1, the better the classification ability of the model. Sensitivity is also an important performance metric, representing the ability of the model to correctly identify actual positive samples (CA events). Developing a highly sensitive model is essential for the CA prediction task because a model with low sensitivity may miss CA events, which could lead to severe consequences for patients. Therefore, the goal of this study was to develop a prediction model with high sensitivity and a low false alarm rate. The false alarm rate is defined as the proportion of incorrectly detected CA events among all alarms. The calculation of the false alarm rate is as follows:

FAR = 1 – (N_True_/N_All_)

where FAR is the false alarm rate, N_True_ is the number of correct alarms, and N_All_ is the total number of alarms.

#### Cross-Dataset Adaptation

We used meta-learning combined with fast adaptation to solve the problem of model adaptation across different datasets, aiming to equip the model with the ability to learn new tasks quickly and adapt to unseen tasks with a small number of samples [20,21]. Specifically, the model was first pretrained on the MIMIC-III dataset to acquire a general learning strategy, thus achieving a good initial state that allows for rapid adjustment when faced with new tasks. Once the model was pretrained, it underwent fast adaptation on the eICU-CRD dataset, where parameters were quickly adjusted using a small amount of data to improve performance on the new dataset. With this approach, the issue of poor generalization capability of existing models was solved, allowing for better adaptation to various clinical settings.

### Experimental Setup

#### Overview

In addition to model evaluation, comparison with baseline models, and cross-dataset adaptation experiments, 3 other sets of experiments were conducted: evaluation of time-phased prediction performance, the impact of feature sets on prediction performance, and the trends of feature changes over time. The subsequent sections will introduce these 3 sets of experiments.

#### Performance Evaluation of Time-Phased Prediction

This experiment evaluated the ability of the model to predict CA 30, 20, and 10 minutes in advance. The primary evaluation metric was sensitivity, which assessed the ability of the model to identify CA events.

#### Impact of Feature Set on Prediction Performance

We constructed 3 types of feature sets: raw features based on vital signs, statistical features, and a combination of raw and statistical features. Various feature set experiments were conducted to determine the impact of each feature set on the performance of the model.

#### Trends of Feature Changes Over Time

We analyzed the average trends of different vital sign values in the CA and non-CA groups over the 30 minutes before the occurrence of CA and visualized these trends.

## Results

### Model Performance Evaluation

This section presents the evaluation results of the model prediction performance, which were evaluated using accuracy, sensitivity, specificity, AUROC, AUPRC, and false alarm rate. Additionally, the performance of the proposed model was compared with that of the baseline models.

**Figure 5:**
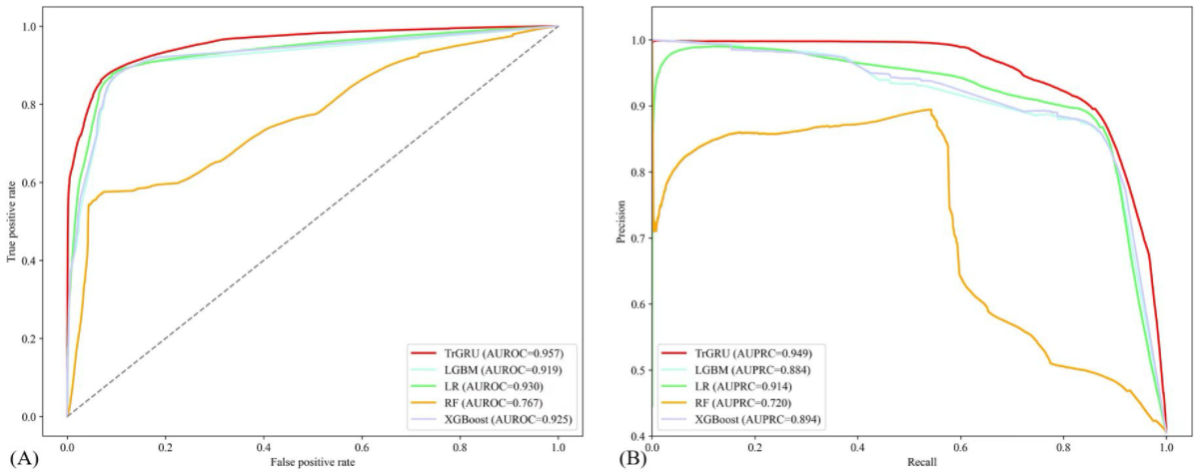
(A) The receiver operating characteristic (ROC) curve and (B) the precision-recall curve on the test set. AUROC: area under the receiver operating characteristic curve; AUPRC: area under the precision-recall curve; LGBM: light gradient boosting method; XGBoost: extreme gradient boosting.

In the MIMIC-III dataset, several models used in existing studies were compared with the proposed model to evaluate its performance in predicting CA events. As shown in Figure 5, the proposed model achieved an AUROC of 0.957 and an AUPRC of 0.949, significantly outperforming the baseline models. In addition, other performance metrics were also compared to fully assess the validity of the model in relevant clinical contexts. As shown in Table 1, the proposed model achieved an accuracy of 0.904, a sensitivity of 0.859, a specificity of 0.933, and a false alarm rate of 0.067, all of which outperform the baseline models, yielding the best performance among these.

**Table 1:** Performance comparison of the proposed TrGRU model with baseline models.

Model	Accuracy (95% CI)	Sensitivity (95% CI)	Specificity (95% CI)	False alarm rate (95% CI)	Area under the receiver operating characteristic curve (95% CI)	Area under the precision-recall curve (95% CI)
Logistic regression	0.895 (0.895-0.897)	0.841 (0.851-0.854)	0.931 (0.925-0.927)	0.069 (0.073-0.075)	0.930 (0.930-0.931)	0.914 (0.913-0.915)
Extreme Gradient Boosting	0.890 (0.889-0.891)	0.852 (0.851-0.854)	0.916 (0.915-0.916)	0.084 (0.084-0.085)	0.925 (0.924-0.925)	0.894 (0.893-0.895)
Light gradient boosting method	0.869 (0.868-0.869)	0.775 (0.773-0.776)	0.933 (0.932-0.934)	0.067 (0.066-0.068)	0.919 (0.918-0.919)	0.884 (0.883-0.885)
Random forest	0.663 (0.662-0.664)	0.708 (0.706-0.709)	0.633 (0.631-0.634)	0.367 (0.366-0.369)	0.767 (0.766-0.768)	0.720 (0.717-0.722)
TrGRU	0.904 (0.903-0.904)	0.859 (0.858-0.861)	0.933 (0.933-0.935)	0.067 (0.065-0.067)	0.957 (0.956-0.957)	0.949 (0.949-0.950)

### External Validation

We used a meta-learning and rapid adaptation–based approach to perform the cross-dataset CA prediction task. On the MIMIC-III dataset, a model was pretrained through multitask meta-learning. After pretraining, the lower-layer parameters of the model were frozen on the eICU-CRD, and only the last 2 fully connected layers were fine-tuned. Domain adaptation was achieved through adaptive training. The experimental results showed that the model achieved a sensitivity of 0.813, an AUROC of 0.920, and an AUPRC of 0.848 on the independent eICU-CRD, demonstrating significantly better cross-dataset adaptation ability compared to current models. These results indicated that the framework effectively used knowledge from the source domain to enhance the few-shot learning performance in the target domain.

### Performance Evaluation of Time-Phased Prediction

We also evaluated the ability of the model to predict CA 30, 20, and 10 minutes in advance, using sensitivity, specificity, AUROC, AUPRC, and the false alarm rate as evaluation metrics, as shown in Table 2.

**Table 2:** Predictive performance of the TrGRU model across different time windows before cardiac arrest (CA).

Before CA	Sensitivity	Specificity	Area under the receiver operating characteristic curve	Area under the precision-recall curve	False alarm rate
30 min	0.906	0.926	0.967	0.955	0.073
20 min	0.926	0.923	0.971	0.958	0.077
10 min	0.948	0.919	0.977	0.963	0.081

The proposed model could identify 90.6% (737/813) of the patients experiencing CA 30 minutes in advance, 92.6% (753/813) of the patients 20 minutes in advance, and 94.8% (771/813) of the patients 10 minutes in advance, with a false alarm rate of 8% or lower within 30 minutes. Compared to other studies, the model proposed in this study exhibited higher sensitivity and a lower false alarm rate.

### Impact of Feature Set on Prediction Performance

We constructed 3 types of feature sets: raw features based on vital signs, statistical features, and a combination of raw features and statistical features. The 3 types of feature sets were input into the model for training to determine the impact of different feature sets on the model prediction performance. The results are shown in Table 3.

**Table 3:** Impact of different feature sets on model prediction performance.

Feature sets	Accuracy	Sensitivity	Specificity	Precision	*F*_1_-score	Area under the receiver operating characteristic curve	Area under the precision-recall curve
Raw features	0.904	0.859	0.933	0.898	0.878	0.957	0.949
Statistical features	0.918	0.894	0.934	0.903	0.898	0.980	0.972
Combination features	0.918	0.892	0.935	0.904	0.898	0.981	0.973

Regarding accuracy, sensitivity, AUROC, AUPRC, and *F*_1_-score, the model performed better when statistical features were used than when raw features were used. When a combination of statistical and raw features was fed into the model, its predictive performance was also superior to that obtained using only raw features. However, its performance showed almost no difference compared to using only statistical features. Consequently, it can be inferred that statistical features enhanced the prediction performance of the model.

### Trends of Feature Changes Over Time

**Figure 6:**
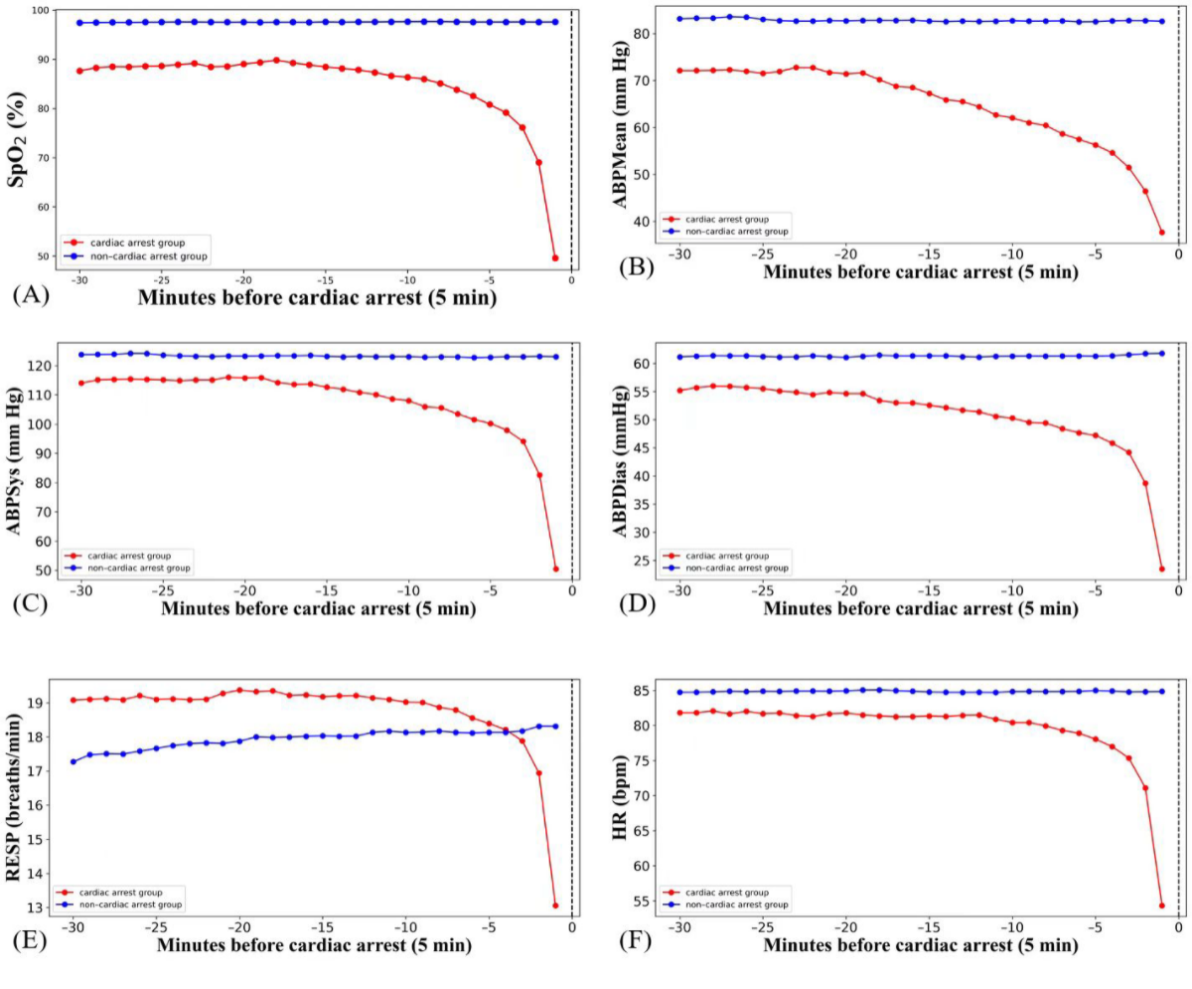
Average trends of vital signs over time in the cardiac arrest and non–cardiac arrest groups. ABPDias: diastolic blood pressure; ABPMean: mean arterial pressure; ABPSys: systolic blood pressure; RESP: respiratory rate; SPO2: oxygen saturation.

Vital sign patterns over time for patients in the CA group and the non-CA group are shown in Figure 6. Compared to the non-CA group, the CA group exhibited lower HR, SpO_2_, and blood pressure values before the event, while respiratory rate was slightly higher. Vital signs began to decline 25 minutes before CA. The decline became more noticeable within the 10 minutes preceding the event. Among all the vital signs, blood pressure and SpO_2_ showed an earlier decline. Compared to the CA group, the vital signs of the non-CA group remained relatively stable.

## Discussion

### Principal Findings

A deep learning model with real-time capability, high sensitivity, and a low false alarm rate was developed using only 6 vital signs based on the MIMIC-III database. The model relied on clinically accessible indicators to determine whether a patient would experience CA within the next hour at 5-minute intervals. It enabled real-time and continuous monitoring by overcoming the limitations of traditional prediction models that rely on a large number of features or features not commonly used in hospitals. The outcomes demonstrated that the model achieved excellent prediction performance. The TrGRU model developed in this study outperformed logistic regression, LGBM, and random forest across all metrics. Notably, compared to other models, TrGRU achieved both high sensitivity and a low false alarm rate. Table 4 presents a comparison between this study and other studies. Layeghian et al [22] developed a prediction model using ensemble learning methods based on multivariate features, vital signs, and clinical latent features, achieving an AUROC of 0.82 on the MIMIC-III dataset. However, their model was not designed for real-time monitoring.

**Table 4:** Comparison with previous studies.

Study	Database	Features	Model	Real time	Cross-validation	Area under the receiver operating characteristic curve
Kim et al [6]	MIMIC-IV	Vital signs, statistical Features, and Gini index	TabNet classifier	Yes	Yes	0.86
Kwon et al [9]	Clinical database	Vital signs	Recurrent neural network	Yes	Yes	0.85
Yijing et al [8]	MIMIC-III	Vital signs	Extreme Gradient Boosting	Yes	No	0.94
Layeghian et al [22]	MIMIC-III	Multivariate, vital signs, and clinical latent	Ensemble learning	No	No	0.82
Our work	MIMIC-III	Vital signs	TrGRU	Yes	Yes	0.96

The experiments investigating the impact of different feature sets on model prediction performance demonstrated that statistical features outperformed raw features in accuracy, sensitivity, AUROC, and AUPRC. When a combination of raw and statistical features was used, performance was also superior to that obtained using raw features alone but comparable to that obtained using only statistical features. Therefore, statistical features played a crucial role in predicting CA events.

Class imbalance is a critical issue that affects model training performance. When the data are severely imbalanced, models often perform poorly in predicting minority classes (ie, they tend to favor predicting negative samples). This situation is particularly common in medical datasets, as in the context of this study, the occurrence of CA events was much rarer than in normal conditions. To address this problem, we used upsampling to replicate positive samples and downsampling to reduce negative samples, thereby balancing the ratio of positive and negative class samples. This solution effectively improved the sensitivity of the model (sensitivity increased from 80.6% before sampling to 85.9% after sampling).

The TrGRU can identify 85.9% (689/813) of the patients 1 hour before a CA event, 90.6% (737/813) of the patients 30 minutes before, and 92.6% (753/813) and 94.8% (771/813) of the patients 20 and 10 minutes before, respectively. These results imply that even when it is too late to prevent CA, medical staff still have a certain amount of time to intervene, thereby improving patient survival rates. This is because the faster cardiopulmonary resuscitation is performed after CA, the greater the patient’s chance of survival, with the survival rate decreasing by 10% per minute before cardiopulmonary resuscitation is initiated. The model proposed by Yijing et al [8] identified 80% of the patients 25 minutes before CA and 93% of the patients 10 minutes before the event. The model proposed by Kwon et al [9] could also only identify 78% of the patients 30 minutes before CA. Therefore, the model proposed in this study demonstrated excellent performance in predicting CA events in advance, outperforming some existing studies.

Due to the “black box” nature of deep learning, it is challenging to establish relationships between real-time prediction results and features. In contrast, TrGRU provided a certain level of interpretability. We analyzed patterns of feature values over time by comparing the changes in vital signs between the CA group and the non-CA group before the occurrence of CA. This provided health care professionals with an objective basis for assessing physiological conditions. Clinical studies have shown that synergistic abnormal changes in vital signs (such as a sharp increase in respiratory rate combined with abnormal fluctuations in body temperature) often exhibit significant characteristic patterns before the occurrence of CA [23-25]. These patterns provide crucial evidence for clinical prediction and the determination of intervention windows.

To improve model generalizability, we used a meta-learning approach to address the issue of the model’s inability to adapt to different datasets. Using the MIMIC-III dataset as the pretraining dataset and the eICU-CRD dataset as the target for rapid adaptation, the pretrained model requires only a small number of samples to quickly adjust and adapt to the new dataset. The results demonstrated that the method proposed in this study significantly improved the generalization of the model’s capability and achieved excellent performance. Kim et al [6] trained their model on the MIMIC-IV dataset and subsequently conducted cross-dataset testing on the eICU-CRD dataset, achieving an AUROC of 0.80. Kwon et al [9] also performed cross-dataset validation on their proposed model, ultimately achieving an AUROC of 0.837 and an AUPRC of 0.239.

### Contributions

This study primarily made 4 contributions. First, compared with existing studies, we developed a highly sensitive CA prediction model. Sensitivity exceeded 90% in predicting CA events within 30 minutes, which could allow health care professionals to intervene earlier and improve patient survival rates. Second, we used a meta-learning approach to enhance the generalizability of the model, enabling it to adapt to different datasets rapidly. To the best of our knowledge, this was the first study to use meta-learning to address the adaptability of medical prediction models across different datasets. Third, the model in this study significantly reduced the false alarm rate, which could effectively help avoid unnecessary interventions or treatments, thereby improving the efficient use of medical resources and reducing health care costs. Additionally, reducing false alarms helps prevent “alarm fatigue” [26], ensuring that health care professionals remain highly alert and responsive to each alarm. Fourth, we developed a prediction model using fewer variables, overcoming limitations of existing models that rely on variables not commonly used in hospitals. This approach offers the advantage of being applicable to various hospital settings.

### Limitations

Our work mainly involves 3 limitations. First, due to the “black box” nature of deep learning, it is unable to establish relationships between prediction results and input data, and the interpretability of results is a critical factor for clinicians in making medical decisions; therefore, deep learning–based medical decision support still faces practical challenges. In recent years, the interpretability of deep learning has been explored in research [27-31], which is a key focus for our next steps. Second, CA may be caused by a variety of diseases, and in this study, we did not consider the heterogeneity between patients with different diseases [32]. Therefore, the generalizability of TrGRU cannot be guaranteed. Accordingly, it is necessary to expand the dataset to cover various types of diseases to further enhance the generalizability and robustness of the model. Third, we did not conduct feature selection. However, based on the research results, it appears that this omission did not significantly affect the performance of the model. In the future, feature selection could be used to further optimize the model presented in this study.

### Conclusions

CA is a sudden and critical event with extremely low survival rates and a poor prognosis, posing a severe threat to patients. Existing CA prediction systems often suffer from low sensitivity and high false alarm rates. Consequently, there is an urgent clinical need for a reliable prediction system to assist health care professionals in real-time monitoring of CA events. The model proposed in this study can accurately predict CA events in real time, with high sensitivity and a low false alarm rate. Additionally, the use of meta-learning significantly enhances the adaptability of the model across different datasets. The TrGRU model shifts the golden resuscitation time window forward for patients who experience CA, which will play a significant role in improving patient survival rates. Moreover, its applicability to different clinical settings facilitates broader adoption.

## Abbreviations

AUPRC: area under the precision-recall curve
AUROC: area under the receiver operating characteristic curve
CA: cardiac arrest
GRU: gated recurrent unit
HIPAA: Health Insurance Portability and Accountability Act
HR: heart rate
ICU: intensive care unit
LGBM: light gradient boosting method
SpO2: oxygen saturation
XGBoost: extreme gradient boosting

## Data Availability

The datasets generated or analyzed during this study are available from the corresponding author on reasonable request.
